# Effects of methamphetamine on two measures of reward: Euphoria and neural activation to reward cues

**DOI:** 10.1038/s41386-025-02110-6

**Published:** 2025-04-23

**Authors:** Hanna Molla, Joseph DeBrosse, Sarah Keedy, Royce Lee, Harriet de Wit

**Affiliations:** Department of Psychiatry and Behavioral Neuroscience, University of Chicago, Chicago, IL USA

**Keywords:** Reward, Motivation

## Abstract

Stimulants enhance dopamine function, affecting diverse aspects of reward function from neural processing of reward cues to feelings of well-being in humans. However, little is known about the relationships among different measures of reward function. Understanding relationships among different indices of reward processing could provide insight into the processes that control motivated behavior. The present study examined the effects of a single dose of methamphetamine on two measures of reward in healthy adults: feelings of well-being and neural activation with reward-related stimuli. In a randomized, within-subject, double-blind study, 88 healthy men and women received a single 20 mg oral dose of methamphetamine (MA) and placebo, across two sessions. Regional activations to reward-related cues were assessed using fMRI during the Monetary Incentive Delay task, and positive subjective effects of MA were assessed using standardized questionnaires. As expected, MA increased euphoria and feelings of well-being. MA had minimal effects on neural activation during either anticipation or receipt of reward, but it significantly increased ventral striatal activation during anticipation of monetary loss, suggesting heightened salience of loss-related cues. As reported previously, caudate activation during reward anticipation during the non-drug (placebo) session was correlated with euphoria induced by MA (on the MA session). However, this correlation between cue-induced neural activation and euphoria was not apparent on the MA session. Thus, MA-induced euphoria was related to reward cue-elicited neural activation only when participants were tested without the drug. MA increased neural reactivity to loss, and this was not correlated with euphoria. These findings suggest that MA can dampen reward-related neural activity normally detected in the drug-free state, and that it enhances brain responses to loss. Further research is needed to determine how neural responses to reward or loss cues are related to feelings of well-being, and how either of these affect reward-related behavior.

## Introduction

A large body of research has implicated dopamine (DA) in the processing of reward and motivated behaviors in both humans and non-human species. Deficits in DA signaling are implicated in psychiatric disorders characterized by impairments in pleasurable experiences, such as depression [1], and activation of DA circuitry is implicated in the rewarding effects of drugs of abuse, including stimulants [2]. However, reward function has been assessed using a wide range of outcome measures, which are sometimes inconsistent [3]. One way to study the role of DA in reward function is using challenge doses of a dopaminergic agent such as methamphetamine (MA) [3]. While MA increases synaptic levels of DA, norepinephrine, and serotonin [4], its effects on reward are typically attributed to its actions on DA. Reward function after administration of MA can be assessed using a range of outcome measures including neural, behavioral, and, in humans, subjective effects. Components of reward function can be further subdivided into many sub-processes, such as anticipation, outcome, pleasure, and reward learning. In the present study, we examined the relationships between two indices of reward function after a single dose of MA in healthy adults: self-reported feelings of well-being and neural responses to cues signaling monetary rewards.

An extensive literature has demonstrated DA’s importance in processing reward-related cues. DA plays a role in both the anticipation of an upcoming reward and during the receipt of reward. The anticipation phase, signaled by a reward-related cue, is associated with reward salience, and implicates brain regions such as the ventral striatum, amygdala, and cortical regions including the anterior insula [5, 6]. The reward receipt or outcome phase, which occurs upon delivery of a reward, is thought to be related to reward appraisal, and is associated with increased activation of the ventral striatum and prefrontal regions including the orbitofrontal and ventromedial prefrontal cortices [5–7]. There is evidence that stimulants increase dopaminergic signals during presentation of cues predicting reward [8–10]. For example [11], used fast-scan cyclic voltammetry to show that low doses of amphetamine increased DA transients elicited by cues predicting the delivery of sugar in rats. In humans, two studies have reported that both *d*-amphetamine and methylphenidate reduced ventral striatal activation in anticipation of a high magnitude monetary reward [12, 13]. In contrast, another study [14] found that the stimulant modafinil increased ventral striatal activation in anticipation of high magnitude reward, and Knutson et al. [13] found that amphetamine increased activation during anticipation of high magnitude loss. In a previous report with a subset of the participants in the present report (*N* = 43) [15], MA increased striatal responses during loss anticipation. In prior studies [13, 15], the effects of stimulant drugs on neural activation are greater during reward anticipation than during reward receipt. Thus, stimulant drugs change neural responses to monetary rewards and losses in humans, but the effects vary across drugs and conditions.

In humans, stimulant drugs like MA also produce subjective feelings of well-being [16–19]. These feelings of well-being are correlated with behavioral measures of drug reward, such as choice of drug versus placebo [20] and conditioned place preference [17]. These behavioral effects resemble findings in homologous animal models of drug reward including self-administration, choice, and place preference [21, 22]. The feelings of well-being and euphoria induced by stimulants in humans have been linked to increases in DA function. For example, A SPECT study with low-dose amphetamine challenge (0.3 mg/kg, i.v.) showed that amphetamine decreased striatal D2 receptor availability, and the magnitude of the decrease was significantly associated with the positive subjective effects of the drug [23]. Similarly, using PET [24] showed that euphoric responses to amphetamine were positively correlated with the magnitude of DA release in the ventral striatum.

Even though the effects of stimulants on both neural processing of reward cues and subjective feelings of well-being in humans have been linked to DA function, few studies have examined the relationship between the two. Two studies [25, 26] examined neural responses to cues predicting monetary rewards in the drug-free state in relation to subjective feelings of well-being after receiving a moderate dose of amphetamine (or placebo). Greater caudate activation during anticipation or receipt of reward in the drug-free state predicted more positive subjective responses to a single dose of amphetamine, suggesting that neural responses to reward cues may be related to stimulant-induced euphoria.

The present study further addressed these questions, by examining effects of MA on neural responses to reward and loss in relation to its effects on feelings of well-being. To assess neural responses to anticipation and receipt of rewards we used the Monetary Incentive Delay task (MID) [13], and feelings of well-being were measured using standardized self-report measures. Healthy young adults (*N* = 88) each received MA (20 mg oral) and placebo in a randomized, counterbalanced design across two laboratory sessions. During these sessions, they completed the MID task in an fMRI scanner and completed self-report questionnaires to assess feelings of well-being. Our primary objectives were (i) to assess the effects of MA on regional BOLD activation during anticipation and receipt of monetary reward during the MID task, and (ii) to determine whether MA-induced neural activation during the task or neural activation to reward cues during the non-drug session, were correlated with positive subjective responses to MA. Based on prior findings [13, 15], we hypothesized MA would induce greater activation in the ventral striatum during monetary reward anticipation, and these changes would be positively associated with drug-induced feelings of euphoria. An exploratory analysis was also conducted to assess the effects of MA on global brain activation patterns during reward anticipation and receipt phases.

## Materials and methods

### Design overview

The study used a double-blind, placebo-controlled design in which healthy participants received MA (20 mg) and placebo during two laboratory sessions with fMRI scans (pre-registered via clinicaltrials.gov, NCT04642820). During the scan, participants completed the Monetary Incentive Delay task, and they completed mood and subjective drug effect questionnaires throughout the sessions. The main dependent measures were (i) activation of reward-related brain regions during anticipation and receipt of rewards in the task and (ii) subjective ratings of euphoria.

### Participants

Healthy adults (*n* = 101; *n* = 88 final; see “Results”) aged 18–35 y/o participated. Screening included physical and mental health exams, electrocardiogram (EKG), and detailed histories of prior drug use. Criteria for inclusion were: right-handedness, normal EKG, BMI between 19 and 26 kg/m^2^, English fluency, and at least a high school education. Applicants were excluded if they had a history of psychosis, severe post-traumatic stress disorder, depression, current suicidal ideation, current severe substance use disorder, taking prescription medications (except birth control), contraindications for MRI, and if they were pregnant, had a history of cardiovascular disease, or consumed more than four alcohol or caffeine-containing beverages per day. Women with normal menstrual cycles (i.e., not taking birth control medications) attended sessions during the follicular phase of their menstrual cycle [27].

### Procedure

First, an orientation session was conducted to explain the study and obtain written consent. The study was approved by the Institutional Review Board of the University of Chicago. Participants were instructed to fast for at least 8 h before the sessions, and refrain from using recreational drugs for at least 48 h, and alcohol for at least 24 h. They were told that their capsules during the sessions might contain placebo, a sedative, or a stimulant. Participants in this study also attended two other sessions (not reported here) without an fMRI scan, in which they received MA and placebo.

The study consisted of two sessions held from 9:00 a.m. to 1:00 p.m., at least 72 h apart. Participants first completed drug screening (CLIAwaived Instant Drug Test Cup), and breath alcohol test (Alcosensor III, Intoximeters, St. Louis, MO), and pregnancy tests (women). After screening tests, mood and cardiovascular measures were obtained (see below). At 9:30 a.m., participants ingested capsules containing MA (20 mg; Desoxyn with dextrose filler) or placebo (dextrose) under double-blind conditions. At 10:30 a.m., further mood and cardiovascular measures were obtained, and participants were escorted to the imaging center for a 75-min MRI scan. During the scan, they completed the MID task. Arterial spin labeling images were acquired to measure cerebral blood flow, but this is not included in the present analysis because we previously [15] found that MA-induced alterations in blood flow were not related to changes in regional BOLD activation. After the scan, participants completed further mood and cardiovascular measures, and participants were discharged at 1:00 p.m.

### Subjective and cardiovascular measures

Subjective (mood) and cardiovascular measures were obtained at baseline (pre-capsule) and 30, 50, 120, 150, 180, and 210 min after capsule administration.

- **Drug Effects Questionnaire** (DEQ) [28, 29]. The DEQ consists of questions on a visual analog scale about the subjective effects of drugs. For the present analyses we used data from the questions “do you feel any drug effect?”, and “do you like the effect?” rated on a 100 mm line from “Not at all” (0) to “Very much” (100).
- **Addiction Research Center Inventory** (ARCI) [30]. The ARCI consists of 49 true/false questions measuring typical drug effects. For this analysis we used two scales: amphetamine-like (ARCI-A) and euphoric effects (ARCI-MBG).
- **End of Session Questionnaire** (ESQ). Participants were asked to guess what drug they thought they received at the end of each session from the following choices: sedative, stimulant, or placebo.
- **Cardiovascular Measures**. Blood pressure and heart rate were monitored using portable blood pressure cuffs (Omron BP791IT, Omron Healthcare).

### Imaging measures

*Monetary Incentive Delay Task* (MID) [31, 32]. The MID was used to assess neural responses to stimuli signaling anticipation and receipt of monetary rewards. During each of 90 trials, participants viewed one of six “anticipatory” cues that signaled upcoming gain (circle) or loss (square) of a varying magnitude (±$5.00, ±$1.00), and neutral cues (±$0.00). Each cue was presented for 2000 ms, with the monetary values shown below the cues. Each cue was followed by a fixation cross for 2000–2500 ms, providing a window to assess neural response to anticipation. Then, a triangle target (150–500 ms) was presented, and participants were told to press a key before disappearance of the target. If they responded before it disappeared, they gained the stated monetary amount during gain trials, or avoided losing that amount during loss trials. If they failed to respond in time, there was no consequence, but the duration of the triangle target was adapted to maintain a maximum hit rate of 66%. The duration of time the target appeared on the screen decreased after repeated successful trials and increased after repeated unsuccessful trials to optimize the hit rate and overall task performance. The reaction times for “hit” trials were recorded and used as a secondary measure of drug effect. A “feedback” stimulus (2000 ms) initiated the feedback period and informed the participant of the trial outcome. Neural responses to the feedback was assessed during this time. Trials were separated by differing intertrial intervals between 2000 and 6000 ms. The 6 trial types (valence [2] x magnitude [3]) were completed 15 times each in randomized order (90 trials in total). To confirm that participants were engaged in the task, we assessed responses to the trials and excluded data for participants with hit rates of less than 44% on at least one of the scans, corresponding to values falling more than 1.5 times below the lowest quartile in the hit rate distribution.

### fMRI data acquisition

MRI data were obtained with a 3T Philips Achieva scanner with a 32-channel head coil. Functional images were acquired using a gradient-echo echo-planar imaging sequence (TR = 2000 ms, TE = 28 ms, Flip angle = 77°; 560 volumes) with 39 axial slices (3 mm thickness, 0.6 mm slice gap) aligned to the AC-PC line, covering a 20 × 20 cm field of view, SENSE factor of 2. The first four volumes were discarded to allow for longitudinal magnetization to reach a steady state. Field maps in opposite phase-encoding directions were acquired prior to the task for distortion correction. High resolution T1 weighted images (MPRAGE sequence) were collected for alignment and spatial standardization of the functional images. Foam padding was used to reduce head movement, and stimuli were projected onto a mirror installed on the head coil.

### First-level functional image processing

AFNI and FSL were used for image preprocessing [33]. The pipeline included EPI distortion correction using FSL’s topup, alignment of the time series to the volume with the fewest outliers, spatial registration of the functional data to the anatomical scan, warping of anatomical data to MNI space and with the transformation applied to functional images, spatially smoothing (5 mm FWHM Gaussian kernel), and intensity normalization. Volumes with >3 mm motion-related displacement were removed. Excessive motion was defined as >50% of TRs censored and these data were excluded. Voxel-wise neural activation was assessed using AFNI 3dDeconvolve, with de-meaned motion parameters, motion derivatives, and white matter signals included as covariates. First-level fixed contrasts examined anticipation and feedback periods [13, 32]. Anticipation phase contrasts were: (1) gain (+$1.00, +$5.00) versus non-gain (+$0.00), and (2) loss (−$1.00, −$5.00) versus non-loss (−$0.00). Feedback (outcome) phase contrasts were: (1) “*hit trials,*” successful trials that result in monetary gain (+$1.00, +$5.00) versus “*miss trials,*” unsuccessful trials with no gain (+$0.00) for gain outcomes, and (2) “*hit trials*,” successful trials that result in avoidance of monetary loss (−$0.00) versus “*miss trials*”, unsuccessful trials resulting in monetary loss (−$1.00, −$5.00) for loss outcomes.

### Statistical analysis

#### Subjective and cardiovascular measures

Subjective and cardiovascular measures were analyzed as a peak change (greatest increase or decrease) from baseline for each session. Peak change scores were compared between MA and placebo conditions using two-tailed paired *t*-tests (SPSS, Version 29). For a more detailed look, euphoria ratings from each time point were included in a repeated measures analysis of variance (RM-ANOVA), with time point and drug condition as within-subject factors. Post-hoc pairwise comparisons were conducted using paired *t*-tests following significant results.

#### MID ROI analysis

Within-subjects analyses compared MA and placebo sessions. During the anticipation phase, predefined regions of interest (ROI) were the anterior insula, caudate, thalamus, and ventral striatum [5, 6]. Spherical masks (8 mm radius) were created based on coordinates from Oldham et al. Mean *t*-values gain vs. non-gain and loss vs. non-loss contrasts were obtained from placebo and MA sessions for each region. ROI’s for the feedback (outcome) phase were amygdala, orbitofrontal cortex/ventromedial prefrontal cortex (OFC/vmPFC), posterior cingulate cortex (PCC), and the ventral striatum [5, 6]. *t*-values were obtained for gain hit vs. miss and loss hit vs. miss contrasts. Repeated measures ANOVAs were conducted with drug as the within-subjects factor (placebo, MA), and sex as a between-subjects factor of non-interest. Effect sizes were reported as partial eta squared (*ƞ*_p_^2^) values.

#### MID whole brain analysis

Whole-brain analyses during anticipation and reward outcome phases were conducted using AFNI’s 3dttest++ (paired *t* tests comparing MA vs. PL activation), controlling for age, sex, and motion. Clusters were significant at *p* < 0.05 FWE-corrected, requiring >13 contiguous voxels (*p* < 0.001 uncorrected). This threshold was determined with 3dClustSim, incorporating subjects’ mean autocorrelation estimates, a gray matter mask, and 3 × 3 × 3 mm voxel resolution.

#### MID reaction time data

MID reaction times were monitored to provide a secondary behavioral measure of drug effect. Hit reaction times during the MID task were assessed using mixed-design ANOVA with drug (placebo, MA), cue magnitude ($0, $1, and $5), and valence (gain trials, loss trials) as within-subject factors and sex as a between-subjects factor of non-interest. We note that stimulus durations were adjusted during the sessions based on subjects’ performance, thus providing a modified index of reaction time.

#### Correlations

For correlations between effects of MA on MID task neural activation and effects of MA on subjective measures, Pearson’s correlations (or Spearman’s rank correlations when scores were non-normally distributed) were conducted using drug-minus-placebo difference scores. Adjustments for multiple comparisons were made using the Benjamani–Hochberg method with a false discovery rate of 5%. Similar correlational analyses were conducted with effects of MA on neural activation and reaction times.

## Results

### Demographics

Of the 101 participants, 13 were excluded from the imaging analysis due to excessive movement (as defined above; 1 subject), poor task engagement on at least one occasion (as defined above; 9 subjects), and technical issues with the MID task during the scan (3 subjects). Most participants were in their mid-twenties, with minimal previous exposure to stimulants (Table 1).

**Table 1:** Demographic and drug use characteristics. Drug use refers to nonmedical use only.

	*n* (%) or mean (SD)
Sex (M/F)	47/41 (53%/47%)
Age	25.2 (4.1)
Race/Ethnicity	
Asian	14 (16%)
Black	3 (3%)
Hispanic/Latino	14 (16%)
White	52 (59%)
Other/More than one	5 (6%)
BMI	23.6 (2.6)
Education (in years)	16.0 (1.6)
Current drug use	
Caffeine drinks per day	1.1 (1.1)
Cigarettes per day	0.2 (0.8)
Alcoholic drinks per week	1.3 (1.1)
Cannabis uses in past 30 days	3.3 (5.6)
Lifetime drug use	
Number of participants who never used stimulants	69 (78.4%)
Mean lifetime uses among those who ever used stimulants *(N* = *19)*	6.7 (13.1)

### Effects of MA on subjective and cardiovascular measures

**Fig. 1:**
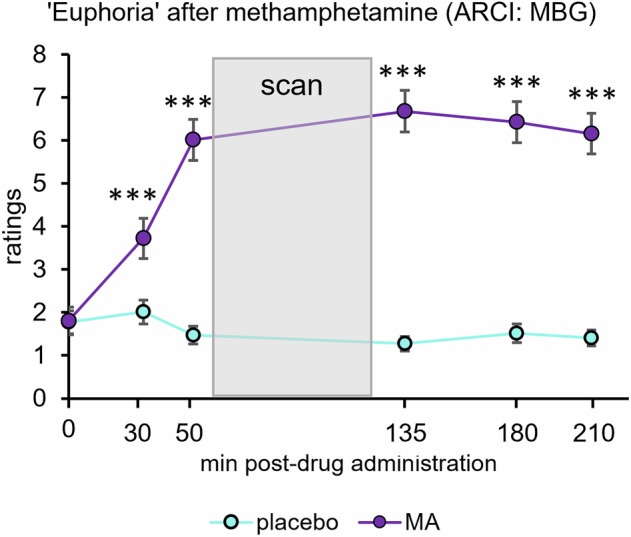
Ratings of Euphoria after MA and placebo. Mean (±SEM) ratings of “Euphoria” ARCI-MBG at specified time intervals after administration of methamphetamine (MA) (purple) and placebo (light blue). Shaded gray bar depicts timeframe when fMRI scan occurred. MA vs. placebo, *p* < 0.001***.

Relative to placebo, MA significantly increased peak change ratings of “feeling a drug effect” (*t(*100) = 9.7, *p* < 0.001) and “liking” the drug effect (*t*(98) = 12.9, *p* < 0.001) compared to placebo. MA significantly increased ARCI “A” scale ratings measuring stimulant-like effects (*t*(100) = 9.1, *p* < 0.001) and the “MBG” scale ratings measuring drug-induced euphoria (*t*(100) = 11.2, *p* < 0.001) (Table 2). Compared to placebo, MA significantly increased Euphoria ratings at multiple timepoints (30, 50, 135, 180, and 210 min post-capsule) (Fig. 1). MA significantly increased heart rate relative to placebo (*t*(100) = 12.5, *p* < 0.001) (Table 2). To assess blinding, participants were asked what drug they thought they received at the end of each session. 73.6% of participants correctly identified receiving a stimulant during the MA sessions, and 59.8% correctly identified receiving placebo during the placebo sessions.

**Table 2:** Mean (±SD) peak change from baseline values for subjective and physiological measures after methamphetamine (MA) and placebo administration.

Peak change from baseline values	Placebo mean (±SD)	MA mean (±SD)	*t*-value	*p* value
DEQ: feel drug	19.1 (21.9)	50.7 (28.4)	9.7	<0.001
DEQ: like drug	23.8 (27.7)	70.5 (29.0)	12.9	<0.001
ARCI: A scale	0.2 (2.1)	4.1 (3.9)	9.1	<0.001
ARCI: MBG scale	−0.03 (3.1)	6.6 (5.7)	11.2	<0.001
Heart rate (bpm)	0.7 (13.4)	23.6 (15.2)	12.5	<0.001

### Effects of MA on reaction time and hit rate during MID task

Regardless of cue valence or magnitude, MA significantly decreased reaction time on hit trials compared to placebo (main effect of drug, *F*(1, 93) = 22.6, *p* < 0.001). Additionally, reaction times were significantly lower during trials with higher magnitude cues ($1 and $5) regardless of valence (gain or loss trials) (main effect of cue magnitude, *F*(1, 93) = 89.0, *p* < 0.001), relative to reaction times for neutral cues ($0) (Fig. S1). Condition order (MA first, PL first) did not have an effect on behavioral responses (main effect of order, *p* = 0.87; interaction effect, *p* = 0.40).

### Imaging

#### MID task activation

Under non-drug (placebo) conditions, “Gain vs. Non-Gain” anticipation cues significantly increased clusters in expected regions, including supplementary motor areas, striatal, and frontal regions (Table S1). On these sessions, feedback cues signaling gains during the outcome phase significantly increased clusters in regions including the putamen and anterior cingulate cortex (Table S1).

#### Effects of MA during anticipation phase of MID task

**Fig. 2:**
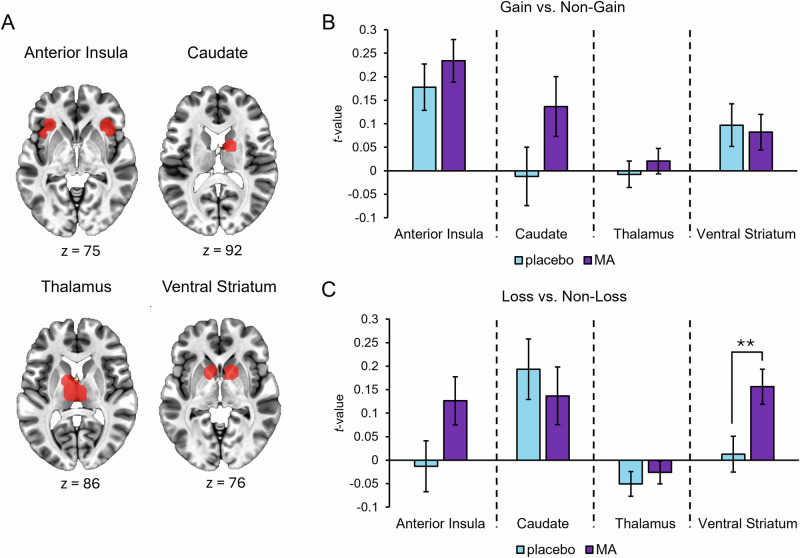
Neural activation after MA and placebo during anticipation components of MID task. **A** Regional masks for the following regions of interest: anterior insula, caudate, thalamus, and ventral striatum, (**B**) Mean ( ± SEM) t-values for BOLD activation during anticipation of gain vs non-gain, and (**C**) anticipation of loss vs. non-loss conditions. Bars represent mean values under placebo (blue) and methamphetamine (MA; purple). MA vs. placebo, *p* < 0.01**.

During anticipation of reward (Gain vs. Non-Gain), there was a trend for greater activation after MA compared to placebo in the caudate (*F*(1, 86) = 3.01, *p* = 0.087, *ƞ*_p_^2^ = 0.034), but the drug did not change activation in other ROI’s including the anterior insula, thalamus, or ventral striatum. During anticipation of loss (Loss vs. Non-Loss) MA significantly increased activation in the ventral striatum compared to placebo (*F*(1, 86) = 7.10, *p* = 0.009, *ƞ*_p_^2^ = 0.076), and non-significantly (trend) increased activation in the anterior insula (*F*(1, 86) = 2.99, *p* = 0.087, *ƞ*_p_^2^ = 0.034). MA did not significantly alter activation in the caudate or thalamus (Fig. 2).

#### Effects of MA during the outcome (feedback) phase of MID task

Relative to placebo, MA did not significantly alter activation in any a priori ROI’s (ventral striatum, amygdala, OFC/vmPFC, and PCC) for feedback cues signaling gains (hits vs. misses) or losses (hits vs. misses) (Table S2).

#### Association between MA-induced neural activation during reward/loss anticipation and subjective or cardiovascular response to MA

As MA did not significantly alter neural responses during the outcome phase, correlations with MA effects were examined only during the anticipation phase. During monetary reward anticipation (Gain vs. Non-Gain), MA-induced BOLD activation did not significantly correlate with subjective ratings. During monetary loss anticipation (Loss vs. Non-Loss), thalamus BOLD activation after MA was positively correlated with stimulant-like effects after MA (ARCI-A scale, *r*_s_ = 0.22, *p* = 0.042; Fig. S2B). However, after applying the Benjamini-Hochberg correction, this effect did not retain statistical significance. Although MA had the greatest effect on BOLD activation in the ventral striatum during anticipation of loss, these effects were not significantly associated with subjective responses.

#### Correlations between MA effects on BOLD activation and MID reaction time

MA-induced changes in activation in any of the studied regions did not significantly correlate with changes in hit reaction times (higher magnitude cue trials vs. neutral trials) following MA administration.

#### Caudate activation during anticipation of reward in the non-drug state as predictor of positive subjective response to MA

**Fig. 3:**
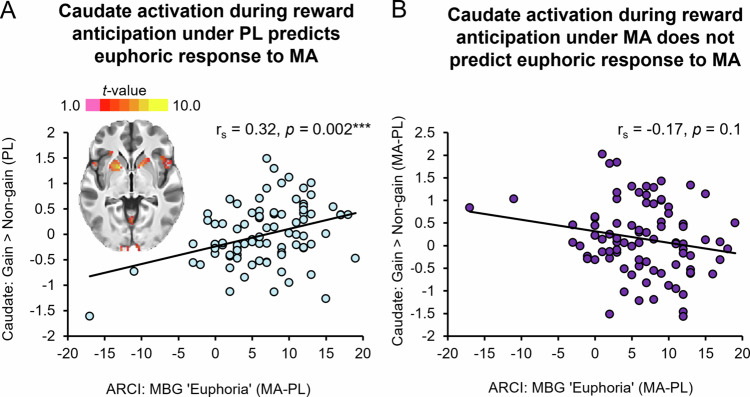
Relationship between caudate activation during the reward anticipation phase and subjective euphoria ratings during placebo (drug-free) and MA sessions. **A** The scatterplot shows a significant positive correlation between R. caudate activation during reward anticipation under the placebo (PL) condition and “euphoria” ratings following methamphetamine administration (MA − PL). Inset brain image shows peak activation (*p* < 0.05, corrected) in bilateral caudate and putamen. **B** The scatterplot shows a non-significant negative correlation between R. caudate activation during reward anticipation under methamphetamine (MA − PL) and MA-induced “euphoria” ratings (MA − PL).

Because a previous study [25] found that caudate activation during reward anticipation *without drug administration* was associated with positive subjective responses to MA, we examined this relationship with the present data. Consistent with the previous finding [25], in the new dataset we also found a significant positive association between caudate BOLD activation during reward anticipation trials in the drug-free state (placebo scan only) and feelings of euphoria after MA (*r*_s_ = 0.32, *p* = 0.002) (Fig. 3A).

#### Correlation between effects of MA on neural activation during anticipation of reward and its positive subjective effects

MA-induced caudate activation during reward anticipation was not significantly related to positive subjective responses to MA (*r*_s_ = −0.17, *p* = 0.1) (Fig. 3B).

#### Exploratory whole-brain analysis

Whole brain analysis revealed no significant effects of MA on cluster activation during anticipation of reward (Gain vs. Non-Gain) or loss (Loss vs. Non-Loss). During the reward outcome (feedback) phase, MA did not alter cluster activation for the hit vs. miss contrast following gain feedback. However, during loss feedback (hits vs. misses), MA increased activation in the right precuneus (44 voxels; *p* < 0.001, *α* = 0.05) relative to PL. This drug-induced change in precuneus activity did not predict subjective responses to MA.

## Discussion

In this study, we examined the effects of a single dose of MA (20 mg, oral) on regional activation during anticipation and receipt of monetary rewards, and their relation to the drug’s pleasurable subjective effects. MA significantly enhanced ventral striatal activation during anticipation of monetary loss, but had little effect during anticipation of wins, or upon receipt of reward (win or loss). Whole brain analyses revealed increased activation in the precuneus after MA during loss feedback. MA produced expected increases in feelings of euphoria and drug-liking. However, the effects of MA on reward-related neural activation were not related to the positive subjective effects of the drug. As reported previously, caudate activation during reward anticipation in the drug-free state was significantly correlated with MA-induced feelings of well-being. However, reward-elicited caudate activation during the MA session was not related to subjective effects of  MA.

The enhanced ventral striatal activation during loss anticipation suggests that MA may increase the salience of signaled losses, perhaps increasing the motivation to avoid an aversive outcome. These findings are consistent with results from Knutson et al.[13], who showed that *d*-amphetamine increased nucleus accumbens activation during anticipation of low and high magnitude loss. They also reported that the drug increased ratings of arousal to loss cues, a measure that was not included in the present study. The ventral striatum is typically known for showing greater activation in response to cues associated with reward, perhaps mediating an increase in the motivation to approach such cues [9, 34, 35]. The present results and those of Knutson suggest that increased monoaminergic function with a stimulant drug may also heighten the salience of loss-related cues. This increased reactivity to loss cues may underlie an increase in motivation to avoid the potential loss, or enhance approach towards “safety” [36]. This increase in reactivity to negative cues may contribute to the effects of MA on decision-making and risk-taking behaviors, particularly in contexts involving aversive cues.

MA had minimal effects on neural activation during the anticipation or receipt of monetary gains. This finding contrasts with one previous report that another stimulant-like drug, modafinil, enhanced reward-related activation in the ventral striatum [14]. It is possible that the MA-induced elevation in synaptic dopamine obscures the neural response to reward-related cues. That is, without the drug, reward-related cues activate  the ventral striatum [9, 37], but after administration of MA, elevated tonic levels of dopamine and other monoamines [4] may obscure the cue-related response [38]. Further studies are needed to identify differences between drugs or doses of drugs that influence the neural responses to reward-related cues.

MA produced its expected rewarding subjective effects, including increased euphoria and drug-liking. However, these subjective effects were not accompanied by an increase in reward-related neural activation after MA. Prior reports (e.g. [12–14]), suggest that MA might either increase or decrease neural response to reward-related cues. The present findings are consistent with the idea that MA increases synaptic levels of DA, which mediates the feelings of well-being but obscures the responses to discrete reward-related cues.

As reported previously, reward cue-elicited activation in the caudate in the drug-free state was correlated with feelings of well-being induced by MA. This suggests that a trait-like measure of reward-related neural re-activity may serve as a neural marker predicting individual differences in the positive subjective effects of MA. That is, individuals with greater caudate activation during reward anticipation may have an inherently greater sensitivity to the drug’s mood-enhancing effects. The finding that this relationship was absent after MA could reflect a ceiling effect of the cue-induced activation, or a masking of the cue-induced activation by increased tonic activity. We note that individuals who showed greater caudate activation to reward-related cues in the non-drug (placebo) condition showed less activation to the cues after MA (*r* = −0.65, *p* < 0.001).

This study had limitations. First, the study used a single, moderate oral dose of MA, and the findings may not generalize to higher doses [39], different routes of administration [40], or to individuals with  other demographic characteristics including chronic MA exposure [12]. Second, the MID task focuses on monetary rewards and losses, which may not fully capture the range of reward-related processes that could be altered by MA. Future research should explore these effects of stimulants using tasks that assess social, emotional, or other types of reward processing.

In conclusion, this study demonstrates that MA selectively enhances striatal activation during the anticipation of monetary loss, while having minimal effects on regional activation during reward anticipation.  Importantly, the study also shows that the euphorigenic effects of MA are related to its effects on neural processing of reward cues in the drug-free state, but not neural responses to reward-cues after MA.  The findings suggest that MA has distinct effects on neural  responses to monetary cues and on subjective feelings of well-being. It remains to be determined whether the neural actions underlying euphoria and processing of discrete cues are truly dissociable, and if so, which actions are relevant to the development of compulsive use and, stimulant use disorders.

## Supplementary information

Supplemental Materials

## Data Availability

Data will be shared for appropriate scientific use upon request.
