# Supplementary material for: Effects of methamphetamine on two measures of reward: Euphoria and neural activation to reward cues
Source: Neuropsychopharmacology. 2025 Apr 23;50(8):1298–304. doi: 10.1038/s41386-025-02110-6 (PMC12170867; doi:10.1038/s41386-025-02110-6)
Supplement: Supplementary file 1 — Supplemental Materials [file 41386_2025_2110_MOESM1_ESM.docx]

**Supplementary Materials**

**Figure S1**

**
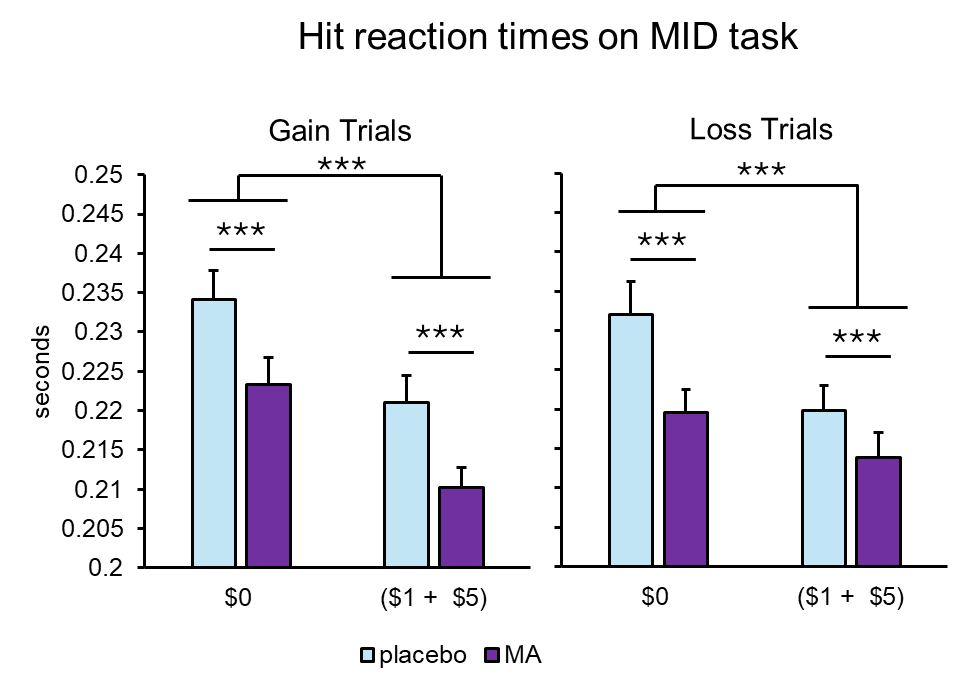
**

**Figure S2**

**A B**

**
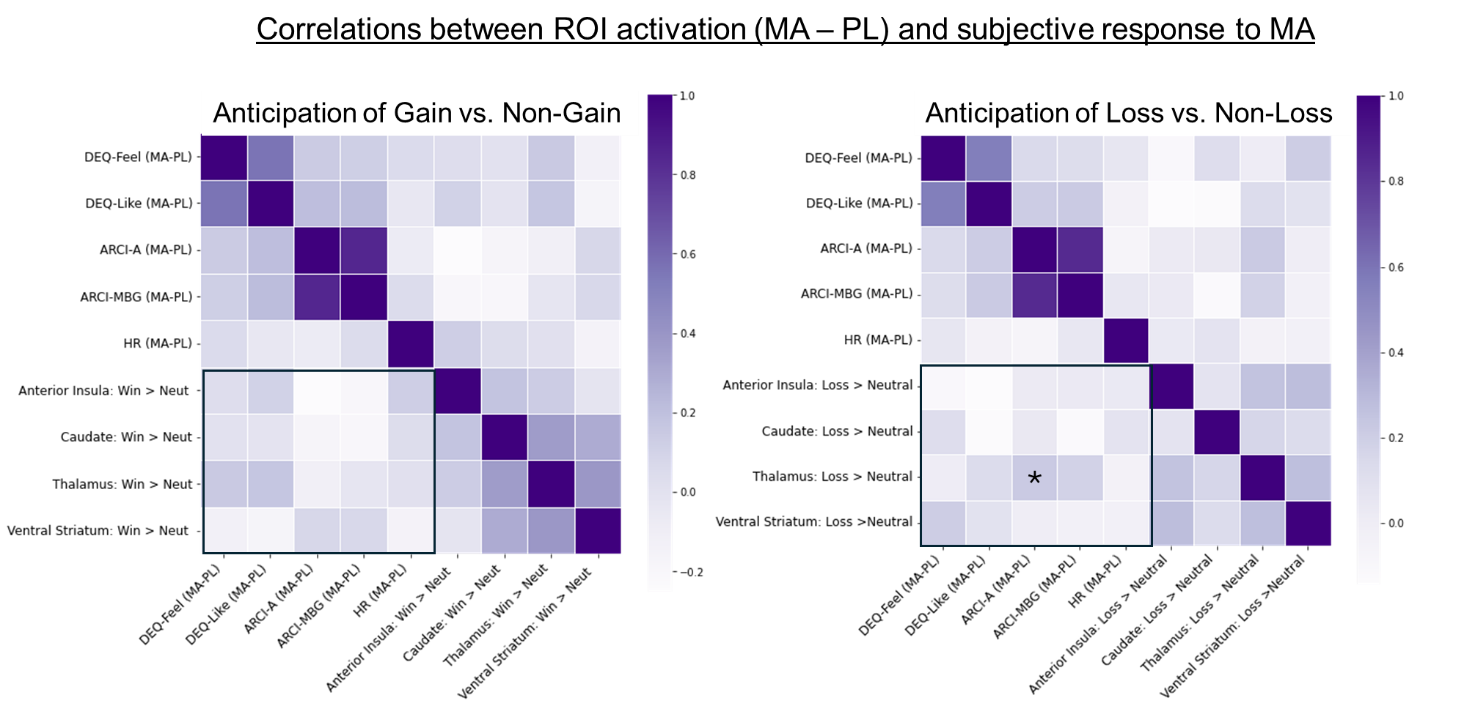
**

Asterisk indicates p < 0.05, uncorrected.

**Table S1.** Task activation during anticipation and receipt of reward on placebo session

|  | MNI coordinates | | |  |  |
| --- | --- | --- | --- | --- | --- |
|  | *x* | *y* | *z* | *t*-value | voxels |
| **Anticipation of gain**  **vs. non-gain** | | | | |  |
| L. SMA | 1.5 | 7.5 | 70.5 | 7.2 | 2822 |
| R. Cerebellum | -31.5 | 49.5 | -31.5 | 5.8 | 1365 |
| L. Putamen | 22.5 | -7.5 | 1.5 | 7.0 | 343 |
| R. Insula | -43.5 | -16.5 | 1.5 | 4.8 | 114 |
| R. Cuneus | -13.5 | 100.5 | 10.5 | 5.4 | 107 |
| R. Cerebellum | -22.5 | 40.5 | -43.5 | 6.0 | 86 |
| R. Caudate | -13.5 | -10.5 | -1.5 | 4.7 | 70 |
| L. Inferior Temporal Gyrus | 49.5 | 1.5 | -43.5 | 4.0 | 60 |
| L. Precentral Gyrus | 55.5 | -4.5 | 40.5 | 3.9 | 48 |
| L. Middle Frontal Gyrus | 31.5 | -61.5 | 16.5 | 4.3 | 45 |
| R. Inferior Temporal Gyrus | -46.5 | 10.5 | -46.5 | 4.9 | 44 |
| L. Posterior Cingulate Cortex | 4.5 | 28.5 | 28.5 | 3.9 | 44 |
| **Receipt of gain vs. non-gain** | | | | | |
| R. Calcarine Gyrus | -16.5 | 94.5 | -1.5 | 5.7 | 397 |
| L. Inferior Occipital Gyrus | 16.5 | 97.5 | -7.5 | 6.8 | 218 |
| L. Putamen | 10.5 | -7.5 | -7.5 | 5.3 | 137 |
| L. Precentral Gyrus | 31.5 | 25.5 | 76.5 | 4.0 | 108 |
| L. Anterior Cingulate Cortex | 7.5 | -43.5 | 1.5 | 4.6 | 86 |
| R. Putamen | -22.5 | -4.5 | -7.5 | 5.1 | 82 |
| L. Superior Frontal Gyrus | 19.5 | -34.5 | 46.5 | 5.2 | 64 |

**Table S2.** *t*-value means (SEM) for ROI’s during the anticipation and outcome phases of the MID

| **Anticipation Phase** | |  | | | |  | | | |  | | | |
| --- | --- | --- | --- | --- | --- | --- | --- | --- | --- | --- | --- | --- | --- |
|  | **Ventral striatum** | | | **Thalamus** | | | **Anterior Insula** | | | | **Caudate** | | |
|  | **placebo** | | **MA** | **placebo** | **MA** | | **placebo** | | **MA** | | **placebo** | | **MA** |
| *gain vs. non-gain* | 0.097 (0.045) | | 0.082 (0.038) | -0.008 (0.028) | 0.02 (0.027) | | 0.178 (0.049) | | 0.234 (0.045) | | -0.012 (0.062) | | 0.137 (0.064) |
| *loss vs. non-loss* | 0.013 (0.038) | | 0.156* (0.037) | -0.050 (0.026) | -0.026 (0.025) | | -0.013 (0.054) | | 0.126 (0.051) | | 0.194 (0.064) | | 0.137 (0.061) |
| **Feedback (Reward Outcome) Phase** | | | | | | | |  | | | |  | |
|  | **Ventral Striatum** | | | **Amygdala** | | | **OFC/vmPFC** | | | | **PCC** | | |
|  | **placebo** | | **MA** | **placebo** | **MA** | | **placebo** | | **MA** | | **placebo** | | **MA** |
| *gain outcome* | 0.260 (0.050) | | 0.207 (0.042) | 0.259 (0.059) | 0.171 (0.051) | | 0.218 (0.068) | | 0.227 (0.059) | | -0.009 (0.086) | | 0.171 (0.073) |
| *loss outcome* | 0.115 (0.050) | | 0.165 (0.036) | -0.043 (0.057) | 0.062 (0.061) | | -0.060 (0.060) | | 0.043 (0.054) | | -0.085 (0.066) | | -0.033 (0.073) |

OFC: Orbitofrontal prefrontal cortex; vmPFC: ventromedial prefrontal cortex; PCC: posterior cingulate cortex

*p* < 0.01*, MA vs. placebo
